# Hydroxyurea Treatment for Sickle Cell Disease

**DOI:** 10.1100/tsw.2002.295

**Published:** 2002-06-25

**Authors:** Martin H. Steinberg

**Affiliations:** Boston University School of Medicine, 88 E. Newton St., Boston, MA 02118, USA

**Keywords:** anemia, fetal hemoglobin, globin genes

## Abstract

High fetal hemoglobin (HbF) levels inhibit the polymerization of sickle hemoglobin (HbS) and reduce the complications of sickle cell disease. Pharmacologic agents that can reverse the switch from γ- to β-chain synthesis — γ-globin chains characterize HbF, and sickle β-globin chains are present in HbS — or selectively increase the proportion of adult erythroid precursors that maintain the ability to produce HbF are therapeutically useful. Hydroxyurea promotes HbF production by perturbing the maturation of erythroid precursors. This treatment increases the total hemoglobin concentration, reduces the vaso-occlusive complications of pain and acute chest syndrome, and attenuates mortality in adults. It is a promising beginning for pharmacologic therapy of sickle cell disease. Still, its effects are inconsistent, trials in infants and children are ongoing, and its ultimate value — and peril — when started early in life are still unknown.

## INTRODUCTION

The new millennium begins an era of remarkable promise for patients with sickle cell disease. Some have longer, more productive lives and their medical costs are reduced because of treatment with hydroxyurea. This review addresses the evolution of pharmacologic treatment for sickle cell disease focused on increasing the concentration of fetal hemoglobin (HbF) in erythrocytes.

More than 50 years ago, infants with sickle cell anemia were noted to have few symptoms during early life, an observation linked to their high HbF levels[1]. Compound heterozygotes for sickle cell trait and gene deletion hereditary persistence of HbF, with more than 20% HbF in all of their erythrocytes, were clinically normal despite having nearly 80% sickle hemoglobin

(HbS)[2,3]. HbF and its γ-globin chains interfere with HbS polymerization; polymer concentrations sufficient to injure the red cell are a prerequisite for causing sickle cell disease. Inhibition of polymerization is localized to the acidic residues, γ80 (Asp instead of β80 Asn) and γ87 (Gln instead of β87 Thr), however other residues could also be important[4]. In erythrocytes, HbS polymer is in equilibrium with HbS monomer whose concentration defines the solubility of HbS. Unlike partially or fully deoxygenated HbS, fully oxygenated HbS is excluded from the polymer phase. If HbF is mixed with HbS, neither the HbF tetramer ($\alpha_2\gamma_2$) nor the $\alpha_2\beta^S\gamma$ hybrid tetramer enters the polymer. In contrast, the hybrid tetramer $\alpha_2\beta^S\beta^A$, has a 0.5 probability of entering the polymer.

Based on these observations, pharmacologic agents were sought that might reverse the neonatal switch from γ- to β-globin chain synthesis[5]. Based on fundamental studies, cytosine hypomethylation was hypothesized to induce γ-globin gene expression, and the first "hemoglobin switching" agent, the nucleoside analog, 5-azacytidine, was postulated to work by causing hypomethylation of the γ-globin gene promoters. This drug increased HbF concentration in sickle cell anemia but was extremely toxic. Cytotoxic drugs incapable of directly causing gene hypomethylation also caused HbF to increase, perhaps indirectly, by perturbing the maturation of erythroid precursors[6,7]. In controlled clinical trials, one of these drugs, hydroxyurea, the principal topic of this review, increased HbF level and improved some symptoms of sickle cell anemia.

## HbF-INDUCING AGENTS

Drugs may increase HbF by several different means; however, their mechanisms of action may overlap, and it is not always clear if a proposed mechanism is responsible for the observed effect.

Three different classes of drugs with the potential to increase HbF concentrations in patients with sickle cell disease and β thalassemia have been the subjects of basic studies and limited clinical trials, but only one drug, hydroxyurea, is used in clinical practice. Before discussing this agent, the other two types of drugs will be briefly reviewed.

### Cytidine Analogs and HbF — An Effect of Gene Hypomethylation?

Hypomethylation at CpG dinucleotides is usually associated with gene expression[8]. Usually, tissue-specific genes are hypomethylated only in the tissues where they are expressed so that, for example, in fetal erythroid tissue γ-globin genes are hypomethylated, while in adult erythroid cells they are methylated[9]. However, hypomethylation does not accompany all expressed genes, and whether or not gene hypomethylation is a primary cause or secondary effect of gene expression remains unclear[8,10]. Incorporation of certain cytidine analogs like 5-azacytidine into DNA inactivates the DNA methyltransferase responsible for gene methylation. These and other preclinical observations led to the use of 5-azacytidine to induce γ-globin gene expression in patients with sickle cell anemia[11,12,13].

5-Azacytidine increased HbF to between 30 and 81% of total hemoglobin in phlebotomized baboons[11]. In sickle cell anemia, 5-azacytidine increased γ-globin mRNA in bone marrow cells, decreased methylation in the γ-globin gene promoter, increased F-reticulocytes, and increased total hemoglobin levels and HbF concentration[12,13,14]. Because of its toxicity, an analog of 5-azacytidine, 5-aza-2'-deoxycytidine, which may have fewer adverse effects, has been studied. Nine patients who failed to respond to hydroxyurea with an increase in HbF synthesis were treated with this compound for 5 days a week over 2 weeks. After 4 weeks of treatment, all patients responded with a mean increase of HbF of nearly 7%, with reversible mild neutropenia the sole adverse effect[15]. While studies are limited, 5-azacytidine may have a more profound effect on HbF levels than hydroxyurea, perhaps because of DNA hypomethylation and its cytotoxicity.

## Short Chain Fatty Acids and HbF — An Effect of Transcriptional Modulation?

Short chain fatty acids like sodium butyrate induce reversible gene expression in cultured cells[16]. Sodium butyrate activated the embryonic ρ-globin gene in adult chicken cells treated with 5-azacytidine, suggesting that gene activation by butyrate may require an "active" chromatin structure[17]. Sodium butyrate is a histone deacetylase inhibitor, a class of agents that may change chromatin structure by causing histone hyperacetylation. When histone deacetylation is inhibited, the N-terminal lysine tails of histones H3 and H4 remain acetylated, favoring euchromatin that can be transcribed[18]. Butyrate may also affect γ-globin gene expression by binding transcriptionally active elements in the 5'-flanking region of the gene[19,20,21].

Sodium butyrate increased HbF in baboons and enhanced γ-globin gene expression in erythroid cells of patients with sickle cell anemia and β thalassemia[22,23,24]. Initial butyrate trials in sickle cell anemia and β thalassemia that continuously infused drug over a 2- to 3-week interval gave inconsistent results, perhaps because of the antiproliferative effects of continuous drug dosing[25,26,27,28]. Butyrate restrains cell growth by inhibiting histone deacetylase, cyclin D1, and through cyclin-dependent kinase inhibitors, cyclin D and E activity[29]. When given only once or twice a month, arginine butyrate was associated with a mean increase in HbF from 7 to 21% in 11 of 15 patients with sickle cell anemia, and in some individuals, this level was maintained for 1 to 2 years[30,31]. Other short-chain fatty acids stimulated HbF production but have not been studied in clinical trials[27,31,32,33,34,35,36] Some of these agents do not inhibit histone deacetylase but stimulate cell growth and also induce HbF expression. This class of compounds has not been evaluated in phase-3 studies. Presently, the use of any short chain fatty acid in sickle cell anemia remains experimental and should be restricted to clinical trials.

## Hydroxyurea and HbF — An Effect of Cytotoxicity?

Between 2 and 3% of circulating erythrocytes of adults are "F-cells", erythrocytes containing detectable HbF[37,38]. Erythroid progenitors often have high levels of HbF[39,40]. Rapid expansion of the erythroid marrow induces F-cell production, suggesting that the kinetics of erythroid regeneration determine whether a red cell will become an F-cell[41]. Early erythroid progenitors may maintain the capacity for γ-globin gene expression, but with differentiation, this "program" is changed, permitting only adult globin gene expression[39,40]. Perhaps earlier progenitor cells contain transacting factors — fetal erythroid Krüppel-like factor is one example[42] — that favor γ-globin gene expression, while late progenitors express other trans-acting factors — erythroid Krüppel-like factor for example — that favor β-globin gene expression[42,43,44]. Cytotoxic agents kill late erythroid progenitor cells, triggering rapid erythroid regeneration and inducing F-cell formation[45]. Accelerated erythropoiesis increases the chance of premature commitment to differentiation resulting in enhanced production of F-cells. Hydroxyurea is an example of a drug thought to increase HbF levels because of its cytotoxicity.

## Pharmacology

Hydroxyurea is a ribonucleotide reductase inhibitor and an S-phase-specific cytotoxic agent. Well absorbed orally, it is converted *in vivo* to a free radical nitroxide that quenches the tyrosyl free radical at the active site of the M2 subunit of ribonucleotide reductase, the enzyme converting ribonucleotides to deoxyribonucleotides[46]. A potent inhibitor of DNA synthesis in cell culture and in organisms from viruses to man, to a lesser degree, hydroxyurea also inhibits RNA and protein synthesis and inhibits DNA repair[47]. Hydroxyurea has a volume distribution equal to body water, with peak serum concentrations reached in 3 to 6 h[47,48]. From 45 to 70% was excreted unchanged in urine, with a variable amount excreted as urea[47]. In the presence of reduced pyridine nucleotide, hydroxyurea was converted to urea in liver and kidney of mice as a direct reduction catalyzed by enzymes present in most subcellular fractions of liver homogenates[49,50]. Besides the urea pathway, hydroxyurea was also reported to be metabolized into genotoxic products by cytochrome P-450–dependent monoxygenases[51]. Hydroxylamine may be rapidly methylated by acetyl-coenzyme A, producing acetohydroxamic acid, a metabolite found in blood of patients given hydroxyurea that may account for up to 10% of the drug administered[52].

Resistance to hydroxyurea occurs by several mechanisms in bacteria and in cell lines. Included are: overproduction of the B2 protein of ribonucleotide reductase; amplification of the M2 gene; increased translational efficiency of M2 mRNA; increased levels of M1 protein; prolonged half life of M1 and M2 subunits; increased M1 and M2 gene copy numbers; production of a resistant mutant enzyme[53]. Similar mechanisms of hydroxyurea resistance have the potential for modulating this agent's effects on erythropoiesis and HbF stimulation.

## Clinical Studies in Sickle Cell Anemia

Hydroxyurea increased HbF level in anemic primates[54]. In sickle cell anemia, hydroxyurea increased F-reticulocytes (HbF-containing immature red cells) and HbF concentration with little short-term toxicity[55,56,57,58]. A multicenter trial of hydroxyurea (MSH) in 299 adults with sickle cell anemia, showed that hydroxyurea reduced the incidence of pain and acute chest syndrome by nearly half; during 9 years of observation, little risk was associated with taking this drug[59].

After 2 years of treatment, adult patients in the MSH had an increase in HbF from a baseline of 5% to about 9%[59]. HbF increased to a mean of 18% in the top quartile of HbF response and to 9% in the next-best quartile, but changed little in the lower two quartiles of HbF response[60]. These results may not reflect what might be expected in all sickle cell anemia patients treated with this drug, since the patients selected for this study were "older" adults who had severe disease and were treated with escalating doses of drug to the brink of myelotoxicity. Not all patients reached the "maximal tolerated dose" specified in the protocol, since the study was stopped prematurely because of the beneficial effects of treatment[61]. Moreover, children appear to have a better HbF response than adults. Perhaps their bone marrow contains more HbF-producing progenitors and is better able to withstand myelotoxicity; they may also be more apt to comply with treatment.

Treatment was associated with less hemolysis, reflected by increased iron clearance, reduced reticulocyte count and plasma iron turnover, and increased total hemoglobin concentration[62]. Some patients had improved physical capacity, with improved anaerobic muscular performance and aerobic cardiovascular fitness[63]. Annual mean costs for painful episodes were $12,000 for hydroxyurea-treated patients and $17,000 for placebo-treated patients. When emergency department visits, costs of analgesics, and blood transfusion were included, the costs were $16,800 for hydroxyurea-treated and $22,000 for placebo-treated patients.

Studies of hydroxyurea in infants, children, and adolescents with sickle cell anemia lag in the rigorous appraisal of clinical efficacy[64,65,66,67,68]. In 93 children and young adult patients treated with hydroxyurea for a median of 3.5 years, the number of hospitalizations and days hospitalized dropped significantly during treatment[69]. About half the treated patients followed for 5 years did not experience any vaso-occlusive events or require hospitalization.

On average, in younger patients HbF increased from 5% before treatment to 16% after 6 months to 1 year of treatment, hemolysis and neutrophil counts decreased, and short-term toxicity was minor[70]. Among 84 children, ages 5 to 15 years (mean age ~10 years), 68 reached the maximally tolerated dose and 52 completed 1 year of treatment[71]. About 20% of enrolled patients were withdrawn from the study, predominantly because of lack of compliance. At baseline, mean HbF was 6.8%, and at the maximally tolerated dose levels increased to 19.8%, with a range of 3.2 to 32.4%[72]. Further analysis of this data suggested that the HbF levels achieved during treatment were associated with baseline HbF level, hemoglobin level, reticulocyte count and leukocyte count, and with treatment compliance[73]. In the MSH, baseline HbF was not associated with HbF levels during treatment, but this study looked at changes in HbF rather than HbF at the maximal tolerated dose[60].

In the youngest children and infants, few results have been reported[74,75]. HbF levels and hemoglobin concentration were increased or maintained. Reliable information on the potential for neurotoxicity and delayed growth in infants with sickle cell anemia given hydroxyurea is lacking, and this absence has hampered studies in this age group. While studies in young mice suggest the possibility of central nervous system damage, it is difficult to extrapolate these results to children; adverse effects on growth and development have not been noticed in children, although the age of patients treated so far may exceed the age of maximal injury potential[76].

We do not presently know whether hydroxyurea will prevent or reverse the organ damage accumulated during the course of sickle cell disease. After 1 year of treatment, splenic function in children with an average age of 12 years did not change[67]. In other studies of children, some appeared to recover or maintain splenic function[75,77]. Splenic regeneration was seen in two adults with sickle cell anemia who had HbF levels of about 30% after hydroxyurea treatment[78,79]. In another preliminary report, sickle pulmonary disease advanced despite a good hematological response to hydroxyurea[80]. Hydroxyurea did not appear to prevent the cerebrovascular complications of sickle cell anemia (see below).

## Clinical Studies in HbSC Disease and HbS-$\beta$ Thalassemia

Pilot studies of hydroxyurea have been carried out in HbSC disease, the second most prevalent genotype of sickle cell disease. Selected hematologic findings in 14 patients with HbSC disease treated with hydroxyurea are shown in Table 1. Cell density fell, as reflected by absolute numbers of erythrocytes and reticulocytes with density >38 g/dl. Pretreatment (0.9 ± 0.8) and final (1.9 ± 2.0%) HbF levels were not statistically different; an increase in HbF from 1.7 to 6.7% in one patient accounted for the difference in the mean HbF level. An increased mean corpuscular volume (MCV), decreased serum bilirubin and reticulocyte count, and an increase in total hemoglobin level suggested reduced hemolysis[81,82]. Twenty-five patients with HbSC disease treated with hydroxyurea have been reported (Table 2)[81,82,83,84]. In one study, patients were selected for severe disease using criteria similar to those of the MSH[84]. In this uncontrolled trial, patients had 21 admissions per year before treatment, compared with 5 admissions per year after treatment. Based on our understanding of the pathophysiology of HbSC disease, reduced numbers of dense cells and a diminished polymerization tendency of HbS should modulate the phenotype of this disease, but no clinical trials have objectively studied the effectiveness of hydroxyurea in this disorder.

**TABLE 1**
**Selected Hematologic Data in Patients with HbSC Disease Treated with Hydroxyurea**

| Patients | PCV | MCV (fl) | rbcHb/rHb | Retic ($10^9$/l) | "Stress" Retic ($10^9$/l) | RBC >1.112 ($10^{12}$/l) |
|---|---|---|---|---|---|---|
| | pre<br>post | pre<br>post | pre<br>post | pre<br>post | pre<br>post | pre<br>post |
| 14 | 32.8<br>34.6 | 75.1<br>84.5 | 28.4<br>39.0 | 177<br>122 | 17.4<br>8.6 | 5.9<br>0.12 |

*Note*: Counts were obtained using an H*3 cell counter. RBC density was measured by phthalate ester gradient centrifugation. Differences between pre- and posthydroxyurea values were all significant ($p$ <.0.05).

**TABLE 2**
**Hematologic Findings in 25 Patients with HbSC Disease Treated with Hydroxyurea**

| Patient No. | Age (years) | PCV | MCV | HbF |
|---|---|---|---|---|
| | | pre<br>post | pre<br>post | pre<br>post |
| 25 | 8-30 | 32<br>34 | 78<br>95 | 1.4<br>5.6 |

*Note*: These data summarize 4 studies of patients of different ages, treated with different doses of hydroxyurea for different time periods.

In HbS-β thalassemia, hydroxyurea appears to work as well as in sickle cell anemia[85,86,87]. In a trial in Greeks with HbS-$\beta^0$ thalassemia and HbS-$\beta^+$ thalassemia, HbF responses were higher than in African-Americans with sickle cell anemia, rising to 23% from the baseline of 3.6%. Twenty-two Sicilians with severe HbS-β thalassemia had an increase in HbF from 7.5 to 25% without reported complications[87]. In both trials, the numbers of patients treated were small and the studies were uncontrolled, but pain episodes were said to nearly stop. Most black patients with HbS-$\beta^+$ thalassemia have 20 to 30% HbA and a mild disease not warranting hydroxyurea treatment.

## *Principles of Treatment*

Indications for hydroxyurea treatment are likely to change as our understanding of drug safety and benefits evolve. Painful episodes that occur at least twice a year and require parenteral opioids, recurrent acute chest syndrome, severe, symptomatic anemia, and perhaps other severe vaso-occlusive complications like priapism are the main indications for starting treatment. Presently, hydroxyurea should be reserved for adults and teenagers with sickle cell anemia or HbS-$\beta^0$ thalassemia who can comply with the treatment regimen and whose complications are sufficiently severe to warrant the burdens of treatment. Severely effected patients with HbSC disease might be given a trial of treatment with the understanding that controlled studies showing a benefit of hydroxyurea in this genotype have not been reported. In young children with sickle cell anemia, outside the context of controlled trials, consultation with expert pediatricians and a detailed explanation to parents of the unknown attributes of this treatment and alternative treatments should precede therapy.

Several reports suggest hydroxyurea as an alternative to transfusion to prevent new or recurrent strokes, especially when transfusions are not feasible[88,89,90]. A child with HbSD disease was treated with hydroxyurea after several strokes and carotid bypass surgery[91]. He reached a HbF level of 30% and, during 28 months of follow-up, had regression of neurologic findings and no further stroke. Of 16 children given hydroxyurea to prevent recurrent strokes, 3 had a new neurological event early during treatment before HbF response was maximized[90]. Other instances of stroke in patients with sickle cell anemia treated with hydroxyurea, who had HbF levels that most would deem "therapeutic", have been recorded[92]. Little information on the distribution of HbF among erythrocytes in these instances — or in most cases of hydroxyurea treatment of sickle cell anemia — or the concentration of HbF/F-cells is available, but in one study F-cells averaged 60% with a high of 80%[68]. If these cells all contained HbF concentrations sufficient to prevent cellular damage, the effects of this level of HbF should be equivalent to a transfusion where 80% of cells are incapable of sickling and only 20% of cells were HbS-cells. Why high HbF levels in hydroxyurea-treated patients with sickle cell anemia do not absolutely protect from stroke is unknown. HbF levels of 20% do not protect absolutely from other disease events, and perhaps stroke is no exception, just a more dramatic example of the shortcomings of a treatment that does not affect the entirety of the pathobiology of sickle cell disease. Alternatively, the pathogenesis of cerebral vasculopathy may differ from that of other vaso-occlusive complications and might not be responsive to the beneficial effects of increasing HbF concentration. Intracerebral hemorrhage, the main cause of stroke in adult sickle cell anemia, occurs at a rate of about 1/400 patient years[93,94]. The MSH trial should be able to detect a doubling in the incidence of intracerebral hemorrhage, but no association of stroke with hydroxyurea was found[95].

Some patients have myelotoxicity at quite low doses, so there is no urgency to arrive rapidly at a final dose. Therapy can be started with 10 to 15 mg/kg given in a single daily dose, or adults can be started on 500 mg daily. With the availability of 200-, 300-, and 400-mg hydroxyurea capsules (Droxia™), it is possible to more precisely titrate the dose of hydroxyurea. Rare adult patients cannot tolerate a 500 mg daily dose. After 6 to 8 weeks, if blood counts are stable, the dose may be increased. Most patients who respond to hydroxyurea maintain tolerable blood counts at doses between 1,000 and 2,000 mg daily (20 to 30 mg/kg). Many different dosing schemes have been recommended, but the sole controlled study gave drug daily and pushed the dose just short of toxicity[61]. Experience has suggested that dose escalation to subtoxic levels is not needed for a satisfactory therapeutic effect in most patients. The therapeutic endpoint should strike a balance between nontoxic doses of hydroxyurea and increases of HbF. Since MCV rises when hydroxyurea is used and usually parallels the increase in HbF, this inexpensive measurement is a useful surrogate for HbF level that can be serially followed. Nevertheless, the concordance between HbF and MCV is not perfect, and HbF should be measured at baseline and 6- to 8-week intervals during the first 6 months of treatment.

Blood transfusions suppress erythropoiesis, and active erythropoiesis is necessary for hydroxyurea to increase HbF. Although not carefully studied, using this agent in patients on frequent or regular transfusion programs is probably not efficacious, although preliminary reports suggested that some chronically transfused children may have a modest HbF increase when also receiving hydroxyurea[96,97].

Until a stable dose is achieved, blood counts should be monitored biweekly. Even when the final dose is reached, counts should be checked at 4- to 8-week intervals to forestall complications from capricious drops in leukocyte or platelet counts, some of which may be unrelated to treatment. Some adult patients will not respond to treatment with an increase in HbF or MCV, even when they faithfully take their prescribed medication, although failure to take the medication regularly seems to account for the largest number of "poor responders"[60,98,99]. True "nonresponders" may number between 10 and 20% of treated patients. A response may depend on the dosing regimen, condition of the bone marrow, genetic determinants, and drug metabolism (see below)[60]. Patients should be explicitly counseled that: not everyone will respond; response differs among patients; many months may be needed to find the best dose of drug; medication must be taken exactly as directed, with frequent blood tests; the long-term toxicities and effects of treatment are unknown[100]. If a patient has not received blood transfusions or had an intercurrent illness that suppresses erythropoiesis, a 6- to 12-month trial should be sufficient to know if hydroxyurea will be an effective treatment, although longer times to response have been noticed.

### *Combination Therapy with Hydroxyurea, Erythropoietin, Other Cytokines, and Butyrate*

Combination chemotherapy, akin to that used in cancer treatment, has the attraction of combining more than one drug, each with a separate effect on HbF synthesis and with nonoverlapping toxicities. Erythropoietin was given with hydroxyurea in nonhuman primates and caused an additive rise in HbF levels[101,102,103]. In a study of two baboons that were made chronically anemic by phlebotomy but were iron sufficient, either hydroxyurea or erythropoietin alone caused a 64 to 283% increment in F-reticulocytes above baseline. Combination therapy with both agents increased F-reticulocytes 150 to 383%[102]. This work suggested that erythropoietin might be combined with agents that cause cytoreduction and rapid erythroid regeneration to achieve a synergistic effect. A study of erythropoietin and hydroxyurea was associated with increases in HbF beyond those seen with hydroxyurea alone[85,104,105]. Four patients with sickle cell anemia who did not respond to butyrate alone were treated with both hydroxyurea and butyrate. Three increased their HbF concentrations from about 1 to 24%[106]. These interesting preliminary results argue for more careful study of this drug combination.

Other cytokines, including c-kit ligand (stem cell factor), GM-CSF, and IL-3 may elevate HbF in primates and in erythroid cell cultures, including cultures derived from patients with sickle cell disease[107], but neither GM-CSF nor IL-3 appear to work synergistically with hydroxyurea. Moreover, a potential to increase greatly the neutrophil count may preclude the use of GM-CSF and IL-3 in sickle cell anemia and in other severe β-globin gene disorders where activated granulocytes may adversely affect the course of disease[101]. When stem cell factor was used in conjunction with hydroxyurea and erythropoietin in baboons, an increment in HbF was observed, compared with stem cell factor and erythropoietin alone[108]. Some treatment regimens caused the leukocyte count to increase acutely and dramatically, so that clinical utility of combinations including stem cell factor is questionable.

*In vitro* studies using a sophisticated method of obtaining sibling BFU-e measured F-cells and γ-globin chains after exposure of erythroid clones to Il-3, GM-CSF, and c-kit ligand. While Il-3 and GM-CSF caused a 2- to 4-fold increase in F-cells and γ-globin chains, c-kit ligand was associated with an increase of 20-fold that was further enhanced by the addition of sodium butyrate[109]. The effect of c-kit on F-cells was accompanied by increased expression of the Tal-1 and FKLF genes that may play a regulatory role in γ-globin gene expression. Transforming growth factor β (TGF β) increased the proportions F-cells during the first 4 days of *in vitro* culture, an increase not dependent on combinations of erythropoietin, c-kit ligand, and IL-3. During continued growth, the number of F-cells in TGF β–treated cultures was approximately tenfold higher than in controls[110].

## Adverse Effects

After more than 9 years of observation, little risk was associated with the careful use of hydroxyurea[95]. Yet, hydroxyurea must be taken indefinitely to be effective and is potentially mutagenic and carcinogenic.

Cellular changes that may antedate neoplastic transformation, like increases in chromosome breakage, recombination events, or mutations in the hprt genes, have not yet been found in hydroxyurea-treated sickle cell anemia patients compared with controls[111]. A study was done of hprt gene mutations and "illegitimate" recombination events between the β and γ gene loci of the T-cell receptor in adults with sickle cell anemia exposed to hydroxyurea for 24 months, children with sickle cell anemia exposed for 7 or 30 months, and adults with myeloproliferative disorders exposed for 11 years. Children who were exposed to hydroxyurea for 30 months had more recombination events (1.8 ± 1.2) compared with children with 7 months of drug exposure (1.6 ± 0.9) and children unexposed to drug (1.1 ± 0.5). No other differences in DNA mutations were found between these groups and unexposed matched controls or normal adults, suggesting to the authors that the leukemogenic potential of the drug is low[111].

Two study patients in the MSH developed cancer[95]. One individual who took hydroxyurea for 63 months had cervical carcinoma *in situ*. Another patient with 47 months of exposure to hydroxyurea had a history of fibrocystic breast disease, and multifocal carcinoma *in situ* was found in prophylactic bilateral mastectomy specimens. Hydroxyurea was used for more than 5 years to decrease PCV in children with cyanotic congenital heart disease without any reports of malignancies, but only 64 patients were followed[112].

Between 5 and 10% of patients with the myeloproliferative disorders polycythemia vera and essential thrombocythemia, who received hydroxyurea, developed acute leukemia[113–120]. This risk rate may not be the same in patients with sickle cell anemia. Two reports of patients with sickle cell anemia treated with hydroxyurea who developed acute leukemia have been published (other cases are known but unpublished) but the total number of patients treated is unknown[121,122]. If the baseline rate of leukemia and cancer in adults with sickle cell anemia is similar to that observed in the general population, a not unreasonable assumption at this time[123,124], because of its size and length of follow-up, the MSH only has power to detect hundredfold increases in the incidence of neoplasia. MSH data suggests that the relative risk of leukemia in patients with sickle cell anemia treated with hydroxyurea is much less than that observed in myeloproliferative disorders and that the risk of death from the complications of sickle cell disease is at least ten times greater than the incidence of leukemia in these patients.

Hydroxyurea is a teratogen in rats, cats, and rhesus monkeys and should not be used if pregnancy is planned. Pregnancy has been reported, in at least 16 women receiving hydroxyurea; most had myeloproliferative disorders but 6 had sickle cell anemia[59,125,126]. Adverse outcomes in this small series did not occur. Contraception should be practiced by both women and men receiving hydroxyurea, and the uncertain outcome of an unplanned pregnancy should be discussed frankly. If a patient who is taking hydroxyurea becomes pregnant, little information is available on which to base a decision for continuation or termination.

Pigmentation of the nails and localized increase in skin pigmentation have been observed and may disappear despite continued treatment[127]. Leg and oral ulcers have been reported in patients with myeloproliferative diseases treated with hydroxyurea[128,129,130,131]. Ankle ulcers were seen when these patients received hydroxyurea for an average of 6 years. They healed when the drug was stopped but recurred when it was restarted. Of 17 adults with sickle cell disease given hydroxyurea for a mean of 3 years, 30% developed leg ulcers, but 80% of these patients had ulcers before[132]. This extremely high incidence of leg ulcers was not seen in the MSH, where hydroxyurea had no effect of on healing or occurrence of leg ulcers. Compared with the observations in myeloproliferative diseases, the follow-up interval in sickle cell anemia on hydroxyurea is short.

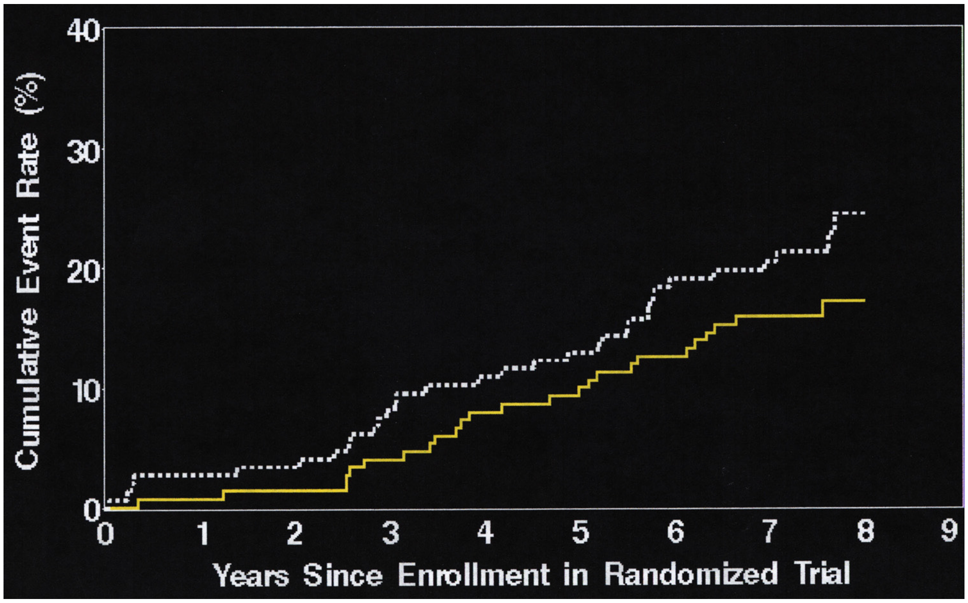

**FIGURE 1.** Cumulative mortality of patients originally randomized to receive hydroxyurea (yellow line) or a placebo (white line). Randomized treatment stopped after 2.5 years. The differences in mortality, when analyzed by original treatment assignment were not significant.

## *Prognosis during Treatment*

Patients in the MSH trial participated in a long-term follow-up study where treatment with hydroxyurea was elective. After nearly 10 years of observation, 25% of patients who volunteered for the MSH had died, reflecting severe disease — a criteria for study enrollment — and the high death rate in adult sickle cell anemia (Fig. 1)[133]. Of these deaths, 30% were due to pulmonary complications. Cumulative mortality was analyzed by (1) original treatment assignment, (2) HbF level, (3) reticulocyte count, (4) hemoglobin concentration, (5) neutrophil count, (6) painful episode frequency, and (7) occurrence of acute chest syndrome. In 278 patients who had HbF measured about 2 years into the MSH, patients with HbF <0.5 g/dl had a 28% cumulative mortality through 9 years, compared with 15% mortality in patients whose HbF was ≥0.5 g/dl (Fig. 2). Before treatment, 68% of hydroxyurea and 65% of placebo-randomized patients had HbF of <0.5 g/dl. When the trial was completed, 38% of the hydroxyurea-randomized patients with initial HbF <0.5 g/dl had HbF ≥0.5 g/dl. In contrast, only 8% of placebo-treated patients with baseline HbF <0.5 g/dL had final HbF ≥0.5 g/dl. Neutrophil counts before random assignment and treatment and at the end of the clinical trial also did not predict mortality. Patients with absolute reticulocyte counts $<2.5 \times 10^5$/ml after 2 years of treatment had a cumulative mortality of 37%, compared with 18% in individuals with $\geq 2.5 \times 10^5$ reticulocytes/ml. In a subgroup of 61 patients with reticulocyte counts $<2.5 \times 10^5$/ml and hemoglobin concentrations <9 g/dl, cumulative mortality was increased after 9 years of observation. These individuals also had lower HbF levels and higher serum creatinine levels and were receiving lower doses of hydroxyurea, compared with other patient groups. Patients without any episodes of acute chest syndrome during the trial had a mortality of 20%, compared with 34% in patients who had one of more acute chest episodes. Individuals with fewer than three annual painful episodes during the clinical trial had a mortality of 19%, compared with 28% in patients with three or more painful episodes annually. Mortality was reduced during 3-month intervals when patients were taking hydroxyurea, from an average of 2.6 ± 5.8 deaths/3-month period to 1.5 ± 7.9 deaths/3 months (Fig. 3).

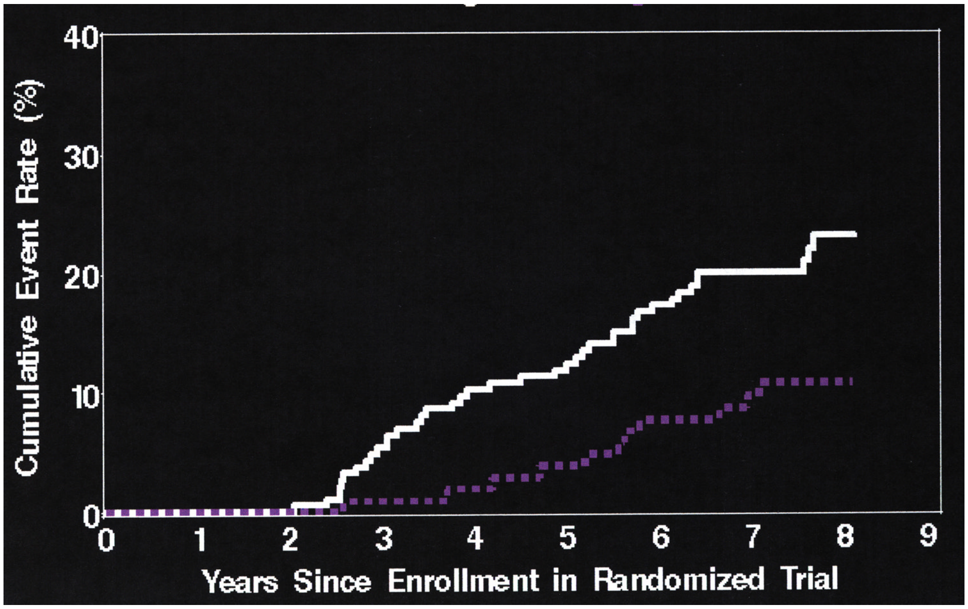

**FIGURE 2.** Cumulative mortality according to HbF levels after the conclusion of the randomized portion of the MSH. White line represents patients with HbF <0.5 g/dL and lavender line patients with HbF >0.5 g/dL ($p = 0.03$).

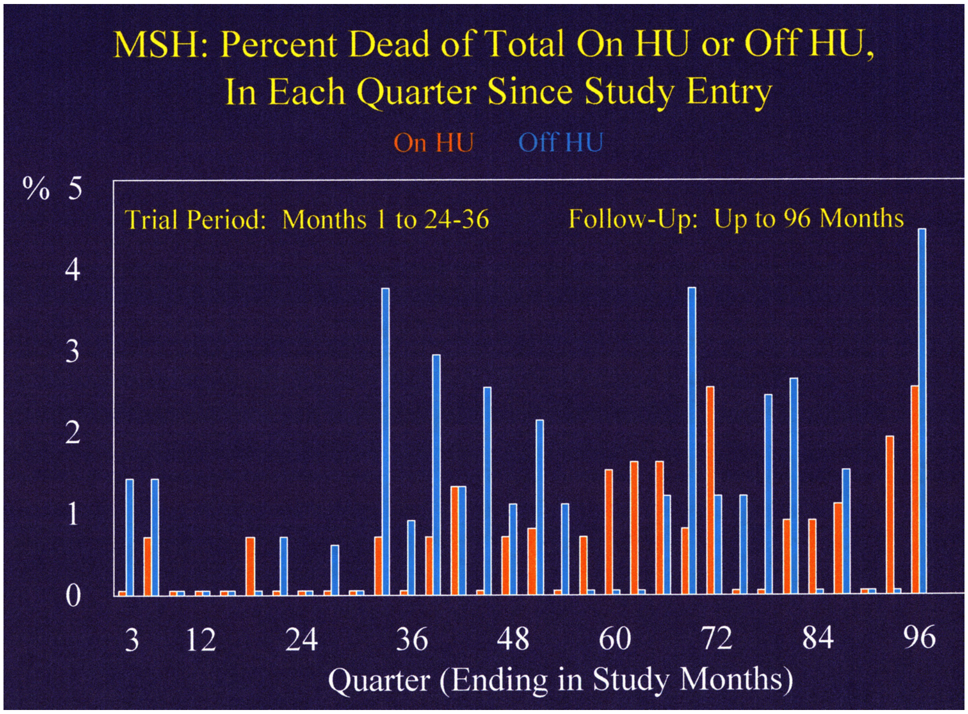

**FIGURE 3.** Mortality by 3-month intervals in patients taking (blue bars) or not taking (orange bars) hydroxyurea. Patients on hydroxyurea had reduced mortality ($p = 0.04$).

These studies suggest that adults with moderate to severe sickle cell anemia who take hydroxyurea have reduced mortality compared with patients not taking this drug. During more than 9 years of observation the estimated overall reduction in mortality was 40%. However, the comparisons of patients on and off hydroxyurea were no longer randomized, and assessment of the effect of hydroxyurea on mortality in an observational study is complex[81]. Nevertheless, the observation of reduced mortality associated with hydroxyurea is consistent with hydroxyurea reducing the incidence of pain and acute chest syndrome by nearly half[59].

## *Non-HbF–Mediated Effects That Might Account for Clinical Effectiveness*

Hydroxyurea may work by multiple mechanisms, although a clinical benefit mediated through its effects on HbF concentration is supported by the strongest evidence. Indisputably, the increase in HbF is a primary effect of treatment, making it difficult to know if the other myriad effects are primary or secondary to increased HbF. While some erythrocyte changes are seen before total HbF concentration is increased, this does not exclude changes in HbF distribution among red cells or small unmeasurable changes in individual cells.

In most open-label trials, improvement in clinical symptoms mirrored — even slightly preceded — the increases in measurable HbF levels. Further studies suggested that the reduction in neutrophils, monocytes, and reticulocytes may also be important[134]. Neutrophils from patients having a painful episode had increased adherence to cultured endothelial cells and expressed higher levels of CD64, an epitope than may promote vascular adherence[135]. By reducing blood neutrophils, hydroxyurea might lessen the chance of vaso-occlusive events.

"Stress" reticulocytes may initiate some vaso-occlusive events of sickle cell disease[136,137,138,139,140,141,142]. A reduction in the numbers of normal reticulocytes and "stress" reticulocytes occurred in sickle cell anemia and HbSC disease patients treated with hydroxyurea, and in sickle cell anemia, this reduction was correlated with the reduction in pain crises. In HbSC disease, hydroxyurea was associated with sustained erythrocyte volume increases and a fall in absolute reticulocyte counts, "stress" reticulocytes, and dense red cells, and this effect seemed to be independent of increases in HbF[81]. During treatment, many simultaneous changes befall the sickle erythrocyte[143]. Sickle cell–endothelial cell adherence decreases before a measured increase in HbF occurs, suggesting a direct effect on red cell membrane or endothelial cell adhesive properties. *In vitro*, hydroxyurea may affect endothelial cells, changing their morphology and cation content and making them a less attractive site for sickle cell adherence[144]. Dense cell numbers fall as erythrocyte $K^+$ content increases[143]. Two reticulocyte adhesion receptors, $\alpha_4\beta_1$ intergrin, or the very late activation antigen (VLA)-4, and CD36, also fall early during hydroxyurea treatment before measurable increases in HbF[145]. Improved cellular hydration and deformability are likely to be secondary to increased HbF and may play a role in the reduction of vaso-occlusive episodes and the reduced hemolysis accompanying treatment[146]. Steady-state soluble VCAM-1 levels are increased in sickle cell anemia compared with normal controls, and a significant decrease was found during treatment with hydroxyurea[147]. Treatment did not affect fibronectin levels or soluble neutrophil adhesion molecules.

*In vitro* studies of the adhesion of sickle erythrocytes to immobilized thrombospondin and laminin showed a 60% reduction in adhesion when patients with sickle cell anemia treated with hydroxyurea were compared with untreated controls[148]. When adhesion before and during treatment with hydroxyurea were compared, treatment was associated with a sustained fall in adhesion.

Other novel mechanisms of action of hydroxyurea have been proposed. Nitric oxide (NO), or endothelial derived relaxing factor, is a potent vasodilator. Peroxidation of hydroxyurea can generate NO[149,150,151]. In rats, hydroxyurea leads to *S*-nitrosohemoglobin formation. Nitrosyl hemoglobin complex could be detected as early as 30 min and persisted up to 4 h after administration of hydroxyurea to patients with sickle cell anemia[152]. In one study, NO increased the $O_2$ affinity of HbS, reducing its polymerization potential, but these results have not been replicated[153,154]. *In vitro* studies have shown that HbS is also able to form sickle nitrosylhemoglobin and that the NO group of this modified molecule derives from the NHOH moiety of hydroxyurea[151]. Normally, *S*-nitrosohemoglobin is formed in the lung, while in tissues, NO is transferred to the vessel wall, promoting vasodilatation[155]. Perhaps hydroxyurea, by promoting NO-mediated vasodilatation, reduces the propensity for microvascular occlusion.

Preliminary studies have shown that hydroxyurea can induce methemoglobin formation and, perhaps, reduce deoxyHbS concentrations, but an early attempt to treat sickle cell anemia by inducing methemoglobinemia was unsuccessful[156,157]. Sickle, but not normal erythrocytes, incubated with hydroxyurea have reduced deformability, an adverse effect proposed to be due to methemoglobin formation[158]. While sickle ghosts and sickle cells incubated with CO were unaffected, methemoglobin levels were not measured.

Hydroxyurea was noted to increase erythropoietin production up to 30-fold over baseline levels 2 to 10 days after beginning treatment. It was hypothesized that this enhances the proliferation of erythroid precursors with the capacity to synthesize HbF[159].

## Predicting the HbF Response to Hydroxyurea

Not every patient responds to hydroxyurea with an increased HbF, and among responders, the HbF increment varies from minimal to more than tenfold[31]. Predicting who will respond to hydroxyurea treatment with an increase in HbF is a worthy goal that is not yet achievable in an individual patient. In 83 hydroxyurea-treated patients using 23 different parameters, correlation and linear regression analysis failed to predict the HbF response, but an artificial neural network pattern-recognition analysis predicted the response with 87% accuracy[160].

In the MSH, the best HbF responses were seen in individuals with highest initial neutrophil and reticulocyte counts and the largest treatment-associated decrements in these counts. Patients with the greatest reduction in granulocyte, monocyte, and reticulocyte counts also had the largest reduction in painful episodes[134]. HbF levels and blood counts changed little from the baseline in poor responders[60]. Most patients responded initially to hydroxyurea with increased numbers of F-cells, but there was a divergence between patients who were long-term responders and those who did not sustain their F-cell response. Among patients with little change in HbF level, neutrophil and reticulocyte counts returned to baseline early during treatment and the percent F-cells decreased after an initial rise. Because of marrow “scarring”, some patients may be unable to tolerate continued myelosuppressive doses of hydroxyurea[161,162].

Myelosuppression may be prerequisite for hydroxyurea-induced enhancement of HbF levels. Patients with the highest baseline granulocyte and reticulocyte counts — who also had the largest decreases in these counts during treatment — had the greatest increases in HbF. In earlier studies, the initial leukocyte count and fall in leukocytes with treatment were also determinants of the final HbF levels after treatment with hydroxyurea[57]. Some patients — perhaps as many as 10 to 20% of all patients treated — did not respond to treatment or had a minor increase in HbF. However, the dosing regimens, final dosages achieved, and length of treatment differed among studies[57,98]. Most patients who respond to treatment will have a response within 1 year of beginning treatment and drug titration.

Genetic elements that may be linked or unlinked to the β-like globin gene cluster may account for differences in HbF level and the HbF response to hydroxyurea. Individuals with the best HbF response were less likely to have a HbS gene on a Bantu haplotype chromosome[134]. The X-linked F-cell production locus phenotype was a determinant of baseline HbF levels, but did not predict the HbF response to hydroxyurea.

A search for polymorphisms in elements linked to the β-globin gene was undertaken to help explain the inconsistency of HbF response to hydroxyurea. Although polymorphisms in elements thought to be important regulators of gene transcription — a 520 base pair core of 5' hypersensitive site (HS)-II of the locus control region (LCR); the $(AT)_x(T)_y$ repeats 5' to the β-

globin gene; a 0.5-kb segment 5' to the $^{G}\gamma$ gene — existed in sickle cell anemia patients with different HbF levels, they and other phylogenetically conserved regions of the LCR were always linked to a particular β-globin gene cluster haplotype[60,163,164,165,166,167]. In "hybrid" haplotype $\beta^{S}$ chromosomes from unselected sickle cell disease patients, the HS-II short tandem repeat (TA)xN10-12(TA)y differed from that typically associated with a cognate haplotype. Each of these "hybrid" chromosomes had a breakpoint upstream of the 0.5-kb pre-$^{G}\gamma$ gene element, suggesting that variability in HbF level associated with polymorphisms of the HS-II enhancer depended on downstream cis-acting elements in tight linkage disequilibrium with HS-II[168]. Conserved noncoding DNA 5' to the β-globin gene cluster may also have important regulatory functions. While hypersensitive sites and their core sequences are conserved among mammalian genomes, percentage-identity plots of the human and mouse β-globin gene clusters showed that sequences outside the cores of hypersensitive sites were as conserved as the cores themselves. These regions also contributed to the ability of LCR DNA fragments to establish and/or maintain an open chromatin domain after stable integration into genomic DNA[169,170,171]. Phylogenetic conservation of some olfactory receptor genes and their flanking sequences 5' to the LCR suggests that this region may also function to control gene expression within the β-globin gene-like complex[172,173,174,175,176].

Twins with sickle cell disease had similar HbF levels whatever their disease phenotype[177,178]. Siblings with sickle cell disease are also likely to share identical β-globin genes with their associated regulatory regions. Therefore, a concordant HbF response to hydroxyurea treatment would suggest that the determinants of this response were, at least partially, linked to this gene cluster. The hematologic response to hydroxyurea was evaluated in siblings from 20 families with sickle cell anemia, including three families with three siblings and six families with HbS-β thalassemia, including one family with three siblings and one pair of monozygotic twins. All patients with HbS-β thalassemia were Greek. A high positive correlation between sibs was found for HbF and mean corpuscular volume (MCV). These observations were independent of the sex of siblings. While other genetic determinants of HbF level unlinked to the β-globin gene cluster exist[179,180,181,182,183], siblings with sickle cell disease are not as likely to share identical transacting factors as they are to share genetic elements linked to their β-globin gene complex. These data suggest that some genetic elements that control the HbF response to hydroxyurea are linked to the β-globin gene cluster. A search for these novel regulatory elements — perhaps in siblings with discordant HbF levels — may identify predictors of the HbF response to hydroxyurea and new targets for pharmacologic or gene therapy.

In summary, compliance with treatment, pretreatment HbF level, bone marrow "reserve", β-globin gene cluster haplotype, and behavior of erythroid precursors in culture may help predict who will have a favorable HbF response to treatment. But currently, none are sufficiently precise to be clinically useful[57,60,73,105,184,185].

## CONCLUSIONS

The phenotype of sickle cell disease is largely dependent on HbS polymerization that, in a complex cascade of pathophysiology, results in damage to the sickle erythrocyte and endothelium. Safely "turning on" HbF genes early in childhood before HbF levels decline to a "subtherapeutic" concentration and vascular damage results, so that each sickle erythrocyte has about 20% HbF, should "cure" sickle cell anemia. Modulation of HbF production using hydroxyurea is a promising beginning for pharmacologic therapy of sickle cell disease. Still, in adults with sickle cell anemia, its effects are inconsistent, and the limit of increasing HbF with this agent alone may have been reached. Combination chemotherapy to increase HbF concentration has a sound conceptual base, but studies of its clinical effectiveness have not yet been done. Agents that prevent or reverse sickle cell dehydration might be added to decrease the cellular concentration of HbS and further retard HbS polymerization[186]. Adding to this, drugs that prevent the abnormal interactions of sickle cells with endothelium would target three of the dominant pathways implicated in propagating the multiple phenotypes of this disease[187].

## BIOSKETCH

**Martin Steinberg** is Director, Center of Excellence in Sickle Cell Disease, Boston Medical Center and Professor of Medicine and Pediatrics, Boston University. Dr. Steinberg is interested in inherited disorders of hemoglobin and how they may be modified by genetic polymorphisms in other genes. He has won numerous awards and honors, including Alpha Omega Alpha, American Society for Clinical Investigation, Association of American Physicians, Central Society for Clinical Research, Southern Society for Clinical Investigation (President, 1993-1994; Founder's Medalist, 2000); Best Doctors in America, $4^{th}$ ed.; and Fellow, American Association for the Advancement of Science.
